# Retraction: Preliminary evaluation of a novel nine-biomarker profile for the prediction of autism spectrum disorder

**DOI:** 10.1371/journal.pone.0312885

**Published:** 2024-11-05

**Authors:** The PLOS ONE Editors

Following publication of this article [1], the following concerns were raised:

- Melatonin concentrations (MLTN) were noted as being considerably higher than would be expected.
- Irregularities were noted in the raw data files for Coenzyme (Q10) and Creatine kinase (CK), suggesting that some of the values may have been duplicated.

Regarding the elevated MTN concentrations, the corresponding author explained that in studies comparing ASD patients and controls, relative elevations or reductions in the levels of the measured variables were more important than the exact values. The corresponding author also suggested that fluctuating melatonin levels could be due to variations in the time of blood collection and potential effects of power of the light–dark cycle, health-related lifestyle factors such as body weight, etc. After a request from a consulting member of the Editorial Board to retest the data using a different kit, the corresponding author explained that they were unable to retest the data as the original testing kit and model were no longer available.

The authors were unable to address the concerns regarding the potential duplications within Q10 and CK raw data, but noted they would be willing to repeat the analysis with Q10 and CK removed.

The concerns raised with the MLTN, Q10, and CK data impact the larger principal component analysis and impact the reliability of the key findings presented in this study. As a result of these concerns the *PLOS ONE* Editors retract this article.

All authors did not agree with the retraction.
